# The phase diagrams of KCaF_3_ and NaMgF_3_ by ab initio simulations

**DOI:** 10.1007/s00269-017-0920-3

**Published:** 2017-09-13

**Authors:** Clément Jakymiw, Lidunka Vočadlo, David P. Dobson, Edward Bailey, Andrew R. Thomson, John P. Brodholt, Ian G. Wood, Alex Lindsay-Scott

**Affiliations:** 1 Laboratoire de Géologie de Lyon, Université Claude Bernard Lyon1, Ecole Normale Supérieure de Lyon, 46 allée d’Italie, 69342 Lyon Cedex 07, France; 2 Department of Earth Sciences, University College London, Gower Street, London, WC1E 6BT UK

**Keywords:** Perovskite, Post-perovskite, Post–post-perovskite, KCaF_3_, NaMgF_3_, High pressure

## Abstract

ABF_3_ compounds have been found to make valuable low-pressure analogues for high-pressure silicate phases that are present in the Earth’s deep interior and that may also occur in the interiors of exoplanets. The phase diagrams of two of these materials, KCaF_3_ and NaMgF_3_, have been investigated in detail by static ab initio computer simulations based on density functional theory. Six ABF_3_ polymorphs were considered, as follows: the orthorhombic perovskite structure (GdFeO_3_-type; space group *Pbnm*); the orthorhombic CaIrO_3_ structure (*Cmcm*; commonly referred to as the “post-perovskite” structure); the orthorhombic Sb_2_S_3_ and La_2_S_3_ structures (both *Pmcn*); the hexagonal structure previously suggested in computer simulations of NaMgF_3_ (*P*6_3_/*mmc*); the monoclinic structure found to be intermediate between the perovskite and CaIrO_3_ structures in CaRhO_3_ (*P*2_1_/*m*). Volumetric and axial equations of state of all phases considered are presented. For KCaF_3_, as expected, the perovskite phase is shown to be the most thermodynamically stable at atmospheric pressure. With increasing pressure, the relative stability of the KCaF_3_ phases then follows the sequence: perovskite → La_2_S_3_ structure → Sb_2_S_3_ structure → *P*6_3_/*mmc* structure; the CaIrO_3_ structure is never the most stable form. Above about 2.6 GPa, however, none of the KCaF_3_ polymorphs are stable with respect to dissociation into KF and CaF_2_. The possibility that high-pressure KCaF_3_ polymorphs might exist metastably at 300 K, or might be stabilised by chemical substitution so as to occur within the standard operating range of a multi-anvil press, is briefly discussed. For NaMgF_3_, the transitions to the high-pressure phases occur at pressures outside the normal range of a multi-anvil press. Two different sequences of transitions had previously been suggested from computer simulations. With increasing pressure, we find that the relative stability of the NaMgF_3_ phases follows the sequence: perovskite → CaIrO_3_ structure → Sb_2_S_3_ structure → *P*6_3_/*mmc* structure. However, only the perovskite and CaIrO_3_ structures are stable with respect to dissociation into NaF and MgF_2_.

## Introduction

If we are to have a proper understanding of the Earth’s mantle, accurate determination of the physical properties of its constituent minerals is essential. For upper-mantle minerals, many of these properties are measureable directly in the laboratory, but the conditions of pressure and temperature that obtain in the lower mantle make such in situ studies increasingly challenging with increasing depth. In particular, experimental determination of the physical properties of the “post-perovskite (or “PPV)” phase of MgSiO_3,_ 1 which is thought to occur just above the Earth’s core-mantle boundary in the D′′ layer, presents a very severe challenge, as it is stable only at pressures in excess of about 100 GPa (see, e.g. Murakami et al. 2004; Oganov and Ono 2004; Tsuchiya et al. 2004; Shim et al. 2008; Tateno et al. 2009). It can be argued that the best way to address these difficulties is via a combination of computational and experimental studies. In this approach, experimental measurements are made on analogue phases, isostructural with the lower-mantle minerals, which are stable (or at least strongly metastable) at ambient pressure and temperature. These measurements may then be used to “ground truth” computer simulations of the analogue phases; if the agreement between experiment and simulations for the analogues proves satisfactory, and the simulations agree with experimental results on the natural system at lower pressures (if available), the computer simulations may then be applied to the natural system at high pressure with a high degree of confidence. As discussed below, two obvious examples of the need for this complementary method come from studies of the cation diffusivities in PPV-MgSiO_3_ (where it has been reported from computer simulations that there is a directional anisotropy of a factor of about 10^8^, Ammann et al. 2010), and in confirming computer simulations of the possible high-pressure phases of MgSiO_3_ under conditions relevant to the mantles of large terrestrial exoplanets (Umemoto et al. 2006a; Tsuchiya and Tsuchiya 2011).

When examining possible lower-mantle analogue phases, fluorides can be considered to have advantages over oxides as their high-pressure phases become stable at much lower pressures. For MgSiO_3_ perovskite (bridgmanite), the mineral neighborite (NaMgF_3_) provides an obvious and attractive analogue (e.g. Umemoto et al. 2006b; Hustoft et al. 2008; Li and Weidner 2012), although other ABF_3_ compounds such as KCaF_3_ (Watson et al. 1995) and KZnF_3_ (Poirier et al. 1983) have also been used for this purpose. NaMgF_3_ may be readily synthesised at atmospheric pressure and is stable at atmospheric pressure and room temperature. At atmospheric pressure and room temperature it crystallises (e.g. Knight 2014), in common with many other compounds (e.g. Mitchell 2002), as an orthorhombically distorted perovskite, in what is commonly referred to as the gadolinium orthoferrite structure (Geller 1956); the space group is *Pbnm* and the system of octahedral tilting producing the distortion from the cubic perovskite aristotype, for this setting of the space group, is a^−^a^−^c^+^ in the notation of Glazer (1972). NaMgF_3_ is, therefore, isostructural with bridgmanite and the two compounds are also isoelectronic; in addition, the relative masses of the atoms in NaMgF_3_ are not too dissimilar from those in MgSiO_3_. It is well known that NaMgF_3_ undergoes a phase transition between the perovskite and CaIrO_3_ structures (Martin et al. 2006a, b) and so this compound may also be used as an analogue for PPV-MgSiO_3_ (e.g. Hustoft et al. 2008). In addition, on the basis of computer simulations, it has been suggested by Umemoto and Wentzcovitch (2006) and by Xu et al. (2015) that further transitions in NaMgF_3_ may occur at higher pressures, which may have some relevance to the mineralogy of super-earths (Umemoto et al. 2006a, b; Grocholski et al. 2010; Tackley et al. 2013). Umemoto and Wentzcovitch (2006) proposed that there would first be a transition from the CaIrO_3_ phase to a lower-symmetry, orthorhombic structure, of the Sb_2_S_3_ (stibnite) structure type (e.g. Lundegaard et al. 2003), as has now been found to occur by experiment in NaFeF_3_ (Crichton et al. 2016) and in NaCoF_3_ (Wood et al. 2017). At still higher pressure, Umemoto and Wentzcovitch (2006) found that a further transition occurred to a hexagonal structure with space group *P*6_3_/*mmc*, which does not seem to correspond to any known structure type; this hexagonal structure was considered by Crichton et al. (2016) to take the Be_3_N_2_ structure (identical to that of InFeO_3_ or InMnO_3_), but although these structures are isopointal (as their space group and occupied Wyckoff positions are the same; Allmann and Hinek 2007), they cannot be considered isoconfigurational as their axial ratios differ by an amount sufficient to cause changes in the primary coordination of the atoms. It should be noted, however, that Umemoto and Wentzcovitch (2006) also concluded that both of these new high-pressure phases would be metastable with respect to dissociation of NaMgF_3_ into CsCl-structured NaF and cotunnite-structured MgF_2_ (a similar sequence of transitions was predicted for CaSnO_3_ by Tsuchiya and Tsuchiya 2006). In contrast, Xu et al. (2015) proposed a different high-pressure transition in NaMgF_3_, from the CaIrO_3_ phase to a structure, with space group *Pnma*, that they termed “ppPv”, for “post-post-Perovskite”. Xu et al. (2015) stated that this structure “has never been previously reported in any material”, but it has since been pointed out by Crichton et al. (2016) that this “ppPv” phase actually corresponds to a structure of the La_2_S_3_ type^2^ (Besançon et al. 1969). Xu et al. (2015) considered that the simulated diffraction pattern of their La_2_S_3_-type structure for NaMgF_3_ was consistent with the “N phase” reported to form at high pressure by Martin et al. 2006a, b, but they did not investigate its stability relative to the Sb_2_S_3_-type structure, nor did they examine its stability with respect to dissociation into NaF and MgF_2_.

In many respects, however, NaMgF_3_ is a much less convenient analogue for PPV-MgSiO_3_ than it is for the PV (bridgmanite) phase. The transition to the CaIrO_3_ structure in NaMgF_3_ occurs at between 28 and 30 GPa (Martin et al. 2006a), which is outside the standard range of a multi-anvil press, and although CaIrO_3_-type NaMgF_3_ can be quenched to atmospheric pressure (Hustoft et al. 2008) some amorphisation of the high-pressure phases on decompression has also been reported (Martin et al. 2006a). For this reason, other ABF_3_ fluorides, such as NaCoF_3_ and NaNiF_3_ (Dobson et al. 2011; Shirako et al. 2012b; Yusa et al. 2012), which may be prepared in a multi-anvil press and then recovered to atmospheric pressure, where they remain quite strongly metastable, have been suggested as more suitable analogues for PPV-MgSiO_3_. These compounds have already proved to be of great value in experimental studies, in revealing the relative strength of PV and PPV polymorphs (Dobson et al. 2012), in demonstrating the topotaxic nature of the PV to PPV transition (Dobson et al. 2013), in determining the degree of anisotropy in the diffusion coefficients of the PPV phase (Dobson et al. 2014) and in allowing the determination of accurate PPV crystal structures (Lindsay-Scott et al. 2014).

A major disadvantage of the ABF_3_ fluorides, where A = Na and B = a first-row transition element, however, is that in the majority of these compounds the *d*-electrons of the B cation will have unpaired spins leading to magnetic ordering at low-temperatures. Bernal et al. (2014) have reported recently that PV- and CaIrO_3_-type NaFeF_3_ show antiferromagnetic ordering at ~90 and 48 K, respectively (with weak ferromagnetism observed in PV-NaFeF_3_); similarly, PV- and CaIrO_3_-type NaNiF_3_ order antiferromagnetically at 156 and 22 K, respectively (Shirako et al. 2012b) and the corresponding phases of NaCoF_3_ have antiferromagnetic transitions at 74 K (Friedman et al. 1970) and 24 K (A S Wills, pers. comm.). Although this magnetic ordering may be of little consequence for experiments carried out at room temperature, i.e. far above the Néel temperature, it does significantly complicate quantum mechanical simulations of these materials, as the correct magnetic ground state must be determined. For this reason we have been considering whether other ABF_3_ compounds, where A and B are cations from groups IA and IIA of the periodic table, can also form CaIrO_3_-type polymorphs at high pressure. PV-structured compounds containing group IA and IIA cations are common, but to date only NaMgF_3_ has been found experimentally to form a phase isostructural with PPV-MgSiO_3_. Considering the most obvious examples, KMgF_3_, KCaF_3_ and RbCaF_3_, it was thought unlikely that either KMgF_3_ (e.g. Wood et al. 2002) or RbCaF_3_ (e.g. Knight et al. 2014) would transform in this way as both are cubic perovskites at ambient pressure and temperature. This supposition is confirmed by studies at high pressure. For PV-KMgF_3_, it was found that the cubic structure is maintained to 50 GPa (Aguado et al. 2008). In contrast, PV-RbCaF_3_ does undergo a lowering of symmetry with increasing pressure, but the transformation, at ~2.8 GPa, is from a cubic to a tetragonal (*I*4/*mcm*) perovskite; no further transformations were observed up to 7.9 GPa, the highest pressure reached in the experiment (Knight et al. 2014).

After NaMgF_3_, KCaF_3_ might appear to be one of the more promising candidate compounds, since at ambient pressure and temperature PV-KCaF_3_ is also orthorhombically distorted with space group *Pbnm*. Furthermore, Fujino et al. (2009) have suggested (for oxides) that for PPV formation, the *t*-factor (Goldschmidt 1926; defined as *t* = (1/√2)(*R*_A_ + *R*_F_)/(*R*_B_ + *R*_F_), where *R*_X_ is an ionic radius) should lie in the range 0.8 ≤ *t* ≤ 0.9; for KCaF_3_, *t* = 0.862, almost identical to the value for NaMgF_3_, 0.866 (ionic radii taken from Shannon 1976). A further requirement (derived from consideration of the behaviour of some A^2+^B^4+^O_3_ perovskites, together with that of NaMgF_3_) was suggested by Tateno et al. (2010) who proposed that for the CaIrO_3_ structure to form, the octahedral tilt in the PV phase at atmospheric pressure and room temperature, *Φ*, estimated from the orthorhombic lattice parameters (in the *Pbnm* setting) by the relationship *Φ* = cos^−1^(√2*a* ^2^/*bc*) (O’Keeffe and Hyde 1977) should be greater than 13°. For these oxides, and for NaMgF_3_, it was found that application of pressure then led to an increase in *Φ*, with the transition to the PPV structure occurring when *Φ* exceeded 19–25°. For NaMgF_3_, *Φ* = 15.0° at atmospheric pressure and 300 K (from the cell parameters given by Mitchell et al. 2007), but for KCaF_3_, *Φ* = 9.3° (Knight et al. 2005). This, however, does not necessarily rule out the possibility of the formation of CaIrO_3_-type KCaF_3_ as: (1) the requirement is only a guide and (2) it is not yet known whether the conclusion that *Φ* should be greater than about 13° is appropriate for fluorides, as opposed to oxides (indeed, the present work suggests that it may well not be, see “Equations of state for KCaF_3_ polymorphs and phase diagram of KCaF_3_”).

It is perhaps surprising that although there have been several crystallographic analyses of PV-KCaF_3_ as a function of temperature (Knight et al. 2005; Mitchell et al. 2007; Knight 2009, 2011), very little has been published on the properties of KCaF_3_ at high pressure. Previous computational work has been confined to investigations of the effect of pressure on ionic conduction in PV-KCaF_3_ by molecular dynamics simulations using interatomic potentials (Watson et al. 1992, 1995) and a recent quantum mechanical study of the effect of pressure on the band structure (Mousa 2014). No experimental high-pressure structural studies have as yet been published, but a number of such experiments have been carried out as part of, or associated with, our present study of KCaF_3_. In the first of these, PV-KCaF_3_ (provided by K S Knight and synthesised as described in Knight et al. 2005) was compressed to 15 GPa and held at this pressure for 24 h at 300 K in a multi-anvil press (MAP) at UCL; on recovery of the sample to atmospheric pressure it was found that it was still composed of PV-KCaF_3_. In further experiments using the MAP at UCL, PV-KCaF_3_ was held at 5.5 GPa and 973 K for 220 min and at 14 GPa and 973 K for 8 h; on recovery to atmospheric pressure and room temperature both samples were found to have decomposed to a mixture of KF and CaF_2_. As mentioned briefly in a paper on RbCaF_3_ by Knight et al. (2014), KCaF_3_ has been studied by neutron powder diffraction up to pressures of 6.86 GPa (at room temperature) on the PEARL beam line at the ISIS Facility of the Rutherford Appleton Laboratory, where it was found that the *Pbnm* perovskite structure was maintained throughout. Similar persistence of the *Pbnm* PV-structure throughout the experiment was also seen in a preliminary examination of KCaF_3_ to ~20 GPa in a diamond-anvil cell (at room temperature) by synchrotron X-ray powder diffraction on beamline I15 at the Diamond Light Source.

In order to determine whether the failure to observe experimentally a CaIrO_3_-type phase of KCaF_3_ was due to an inherent lack of stability or was merely a consequence of the pressure and temperature range covered in these few experiments, it was decided to supplement them with ab initio computer simulations. In this way we have been able to examine the relative stabilities in KCaF_3_ of: (a) the four ABX_3_ polymorphs described by Umemoto and Wentzcovitch (2006) for NaMgF_3_, (b) the “ppPv” (La_2_S_3_) structure reported by Xu et al. (2015), (c) the monoclinic phase found by Shirako et al. (2012a) as an intermediate between the PV and CaIrO_3_ structures in CaRhO_3_, and also to determine the stability of these KCaF_3_ compounds relative to a mixture of KF and CaF_2_. We have found that the PV phase of KCaF_3_ is, as expected, the stable phase at atmospheric pressure but that at pressures in excess of about 2.6 GPa, none of the KCaF_3_ structures are thermodynamically stable with respect to a mixture of KF and CaF_2_. In addition, as the sequence of phase transitions in NaMgF_3_ predicted from ab initio simulations by Umemoto and Wentzcovitch (2006) and by Xu et al. (2015) differed, we also took this opportunity of carrying out a similar study of NaMgF_3_; we find that in NaMgF_3_ the PV and CaIrO_3_ structures are thermodynamically stable, but that none of the proposed higher-pressure phases are stable with respect to a mixture of NaF and MgF_2_.

## Computational method

The static ab initio calculations presented here used the projector augmented wave implementation (Blöchl 1994; Kresse and Joubert 1999) of the density functional theory (Hohenberg and Kohn 1964; Kohn and Sham 1965) with the generalised gradient approximation (GGA) PBE pseudopotentials (Perdew et al. 1996) implemented in the VASP computer code (Kresse and Furthmüller 1996; Kresse et al. 2010). For KCaF_3_, an electronic minimization convergence criterion of 10^−6^ eV was used for the internal energy, with a *k*-point density of 4 × 4 × 4 (Monkhorst and Pack 1976) and a kinetic energy cutoff of 500 eV. For NaMgF_3_, the same electronic minimization convergence criterion for the internal energy was used, but (for consistency with earlier work; Lindsay-Scott 2012) the *k*-point density was increased to 6 × 6 × 6 (Monkhorst and Pack 1976) and the kinetic energy cutoff to 900 eV.

The procedure adopted to determine the volumetric equation of state, and hence the enthalpy of each phase, was to use VASP to calculate the internal energy (*E*) of the crystal at a set of chosen volumes (*V*), allowing the cell parameters and the fractional coordinates (where necessary) to relax in accordance with the crystal symmetry. All simulations were made along a path of decreasing volume. During the calculations the program parameters were set such that the symmetry of the crystal was maintained. Transitions to structures with lower symmetry were, therefore, forbidden but transitions to structures whose space groups are supergroups could occur, as the atoms are not prevented from moving into a more symmetrical arrangement. Since the calculations were static and effectively equivalent to *T* = 0 K, the pressure (*P*) at any point on the *E* vs. *V* curve is given by the standard thermodynamic result *P* = −(∂*E*/∂*V*)_*T* = 0_ (see, e.g. Pippard 1966), the actual values being determined by fitting the *E* (*V*) curve to an integrated Birch-Murnaghan 3rd-order equation of state (EoS; see, e.g. Vočadlo et al. 1999). Knowing *P*, *V* and *E*, the enthalpy, *H*, may be calculated. Since *T* = 0, the enthalpy is equal to the Gibbs free energy, *G*, and thus the most stable phase at any given pressure may be determined.

## Results and discussion

### General considerations

**Fig. 1:**
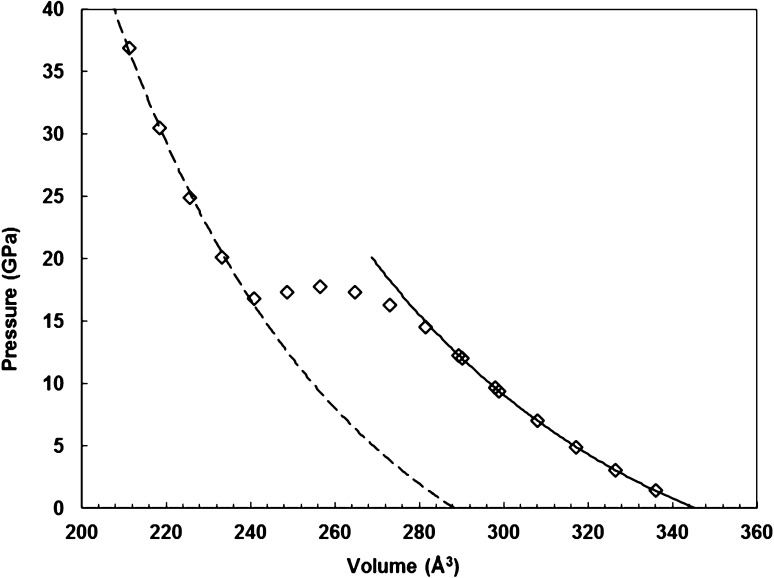
Pressure versus volume for KCaF_3_ obtained by compression of the *Pbnm* perovskite structure. The pressure values at the simulation points (*symbols*) are taken from the VASP output; the lines show Birch–Murnaghan 3rd-order equations of state, fitted to *P* (*V*). The transition from the PV to the *P*6_3_/*mmc*-NaMgF_3_ structure can be clearly seen

Initially, it was intended in this study to consider only the relative stabilities of the PV- and CaIrO_3_-phases of KCaF_3_. It was found, however, see Fig. 1, that when the volume of the unit cell of PV-KCaF_3_ was reduced below about 280 Å^3^ (~17 GPa) a spontaneous transition occurred in the simulations, leading to a different crystal structure. Inspection of the resulting fractional coordinates, using the crystal structure plotting package *Diamond* (Putz and Brandenburg 2006), revealed that this structure corresponded to the hypothetical high-pressure hexagonal phase of NaMgF_3_ (space group *P*6_3_/*mmc*) previously described by Umemoto and Wentzcovitch (2006); in this structure, which does not appear yet to have been observed experimentally, the B cations are [8]-coordinated by the anions. The occurrence of this transition then led us to consider three further possible structures, as follows: (1) the Sb_2_S_3_—structured phase of NaMgF_3_ with space group *Pmcn* (Umemoto and Wentzcovitch 2006), containing B cations sevenfold coordinated by the anions (as observed recently in NaFeF_3_ and NaCoF_3_ above ~22 GPa in synchrotron X-ray diffraction experiments at room temperature by Crichton et al. 2016 and Wood et al. 2017); (2) the La_2_S_3_-structured phase found by Xu et al. (2015); (3) the monoclinic (*P*2_1_/*m*) “intermediate phase” reported by Shirako et al. (2012a) for CaRhO_3._ 3 The PV, Sb_2_S_3_ and La_2_S_3_ structures are isosymmetric, but in order to more readily understand the relationship between the structures, it is convenient to use the *Pbnm* setting of their space group for the PV phase and the *Pmcn* setting for the Sb_2_S_3_ and La_2_S_3_-structured phases; the space group of the CaIrO_3_ structure is *Cmcm*. The symmetry relationships between these structures are interesting in that *Cmcm* is a supergroup of *Pbnm* and thus symmetry is increased in a PV to CaIrO_3_-type transition; the symmetry then reduces on transformation to either the Sb_2_S_3_ or La_2_S_3_-phases, but rises once more in the *P*6_3_/*mmc*-NaMgF_3_ structure as *P*6_3_/*mmc* is a supergroup of *Cmcm* (and thus also of *Pmcn*).

**Fig. 2:**
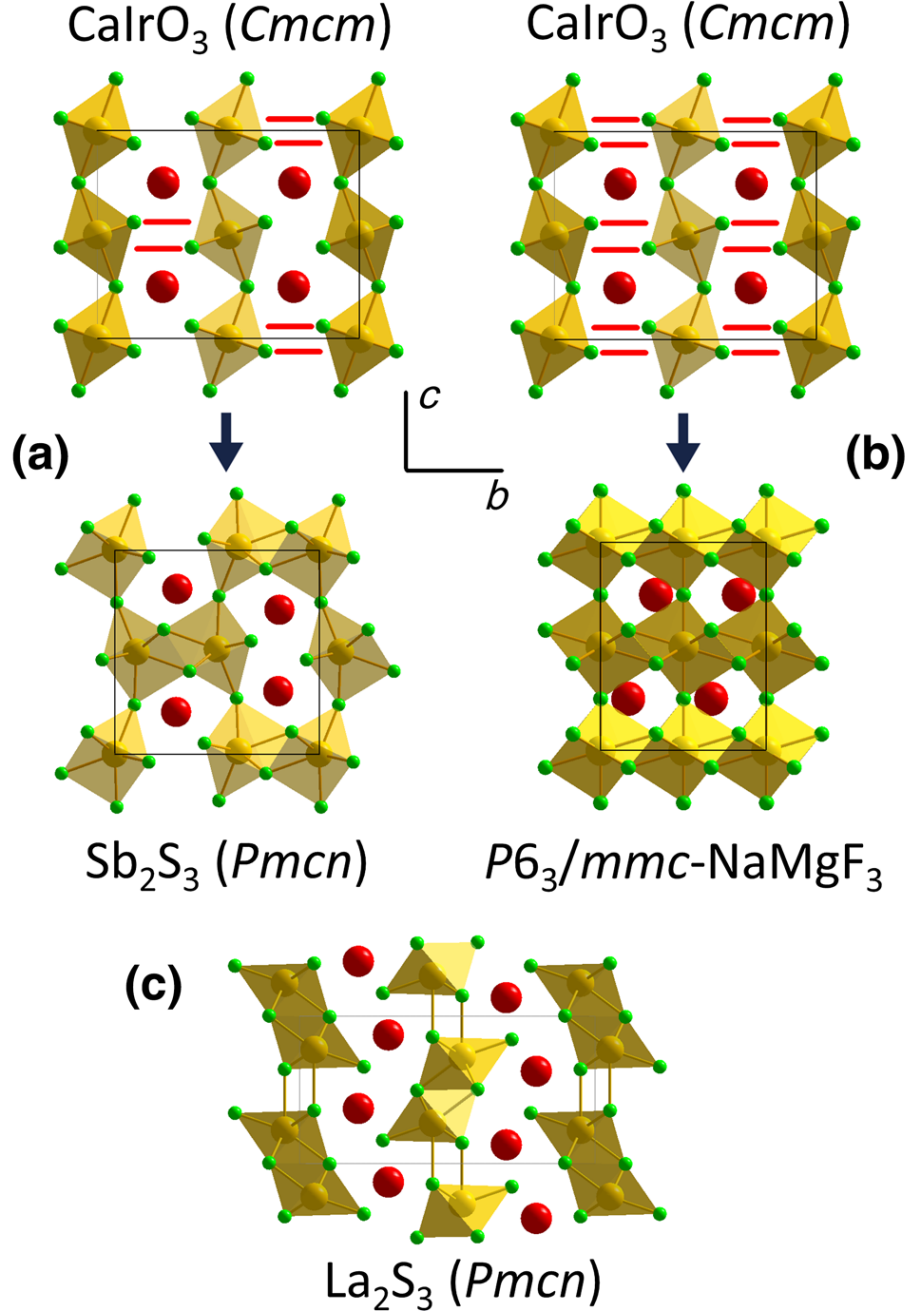
The structural mechanism for the transformations between **a** the CaIrO_3_ and Sb_2_S_3_ structures and **b** the CaIrO_3_ and *P*6_3_/*mmc*-NaMgF_3_ structures (after Umemoto and Wentzcovitch 2006); **c** the La_2_S_3_ structure of NaMgF_3_ (coordinates from Xu et al. 2015, transformed to the *Pmcn* setting). The A-cations are shown in red, the B-cations in gold and the anions in green. The structures are viewed along the orthorhombic *a*-axis, with the orthorhombic *b*- and *c*-axes as marked; so as to enable ready comparison with the CaIrO_3_ and Sb_2_S_3_ structures, the hexagonal *P*6_3_/*mmc*-NaMgF_3_ structure is represented here using a *C*-centred unit cell with an orthorhombic metric in which *b* = √3a. In **c** the coordination polyhedra are drawn as octahedra; there is a 7th Mg–F distance nearly parallel to the *c*-axis (shown here as a *bond*) which is only slightly longer; for details see text

The structural relationship between the CaIrO_3_, Sb_2_S_3_ and *P*6_3_/*mmc*-NaMgF_3_ phases in an ABF_3_ compound is readily understood from Fig. 2a, b (after Umemoto and Wentzcovitch 2006). Starting from the CaIrO_3_ structure, the Sb_2_S_3_ structure can be envisaged as forming with increasing pressure in the following way. The CaIrO_3_-type structure contains two-dimensional sheets of edge and corner linked BF_6_ octahedra lying perpendicular to the *b*-axis. As the *b*-axis (the most easily compressible of the three, see Table 2) shortens on compression, F^−^ ions enter the primary coordination shell of B ions in adjacent sheets of octahedra, thereby linking alternating pairs of BF_6_ octahedra, and increasing the primary coordination of all of the B cations from 6 to 7 (Fig. 2a). The *P*6_3_/*mmc*-NaMgF_3_ structure then forms by a similar mechanism, whereby further reduction of the axial ratio *b/a* to √3 and linkage of all octahedra (rather than just alternating pairs) by F^−^ ions from adjacent sheets leads to a structure with hexagonal symmetry and B ions in [8]-fold coordination by F (Fig. 2b).

The transition between the CaIrO_3_ and La_2_S_3_-structures (Xu et al. 2015) is quite different in that the sheets of edge and corner linked BF_6_ octahedra found in the CaIrO_3_ structure are disrupted. With space group setting *Pmcn* for the La_2_S_3_ phase of NaMgF_3_, there is a direct correspondence between the axes of the unit-cell of these two structures. Xu et al. (2015) found that on forming the La_2_S_3_ structure the *b*-axis of CaIrO_3_-type NaMgF_3_ *expanded* by ~25%, whilst the *c*-axis *contracted* by a similar percentage; the *a*-axis shows about a 4% expansion. It is, therefore, difficult to envisage formation of the La_2_S_3_ phase by hydrostatic compression of a CaIrO_3_-type structure in a real material, as in all such structures for which axial incompressibilities have been measured to date (e.g. Boffa Ballaran et al. 2007; Lindsay-Scott et al. 2010; Wood et al. 2017) the *b*-axis has been found to be considerably more compressible than either *a*- or *c*-axes. Figure 2c shows the La_2_S_3_ structure, which can perhaps be considered to form in the following way. As the *c*-axis shortens, the tilt of the corner-linked octahedra (the angle Δ_1_ in Lindsay-Scott et al. 2011) increases until a point is reached at which the sheets of octahedra cannot be maintained and alternating double rods of edge-linked octahedra running parallel to the *a*-axis are formed. Whether these rods are then considered to be linked along the *c*-axis, depends on whether the B cation is considered to be in sixfold or sevenfold coordination; from the coordinates published by Xu et al. (2015), we find 6 Mg-F distances in the range 1.81–1.94 Å and one slightly longer distance—the Mg–F bond closely parallel to the *c*-axis in Fig. 2c—of 2.10 Å.

### Equations of state for KCaF_3_ polymorphs and phase diagram of KCaF_3_

Table 1 shows the volumetric equation of state (EoS) parameters, derived from fitting *E(V)*, for the PV, *P*2_1_/*m*-CaRhO_3_, CaIrO_3_, La_2_S_3_, Sb_2_S_3_ and *P*6_3_/*mmc*-NaMgF_3_ phases of KCaF_3_, together with those for the relevant phases of KF and CaF_2_. In the pressure range of interest, 0 to ~30 GPa, KF undergoes a transition from the NaCl to the CsCl structures (Weir and Piermarini 1964); similarly, the parameters for CaF_2_ in both the low-pressure fluorite and high-pressure cotunnite structures (Wu et al. 2006) are given. The axial EoS parameters for the non-cubic phases, derived from fitting 2nd-order Birch–Murnaghan EoS to the VASP output pressure versus volume, using the program EoSFit (Angel 2001) are given in Table 2. To enable ready comparison with the volumetric EoS, the values of *K*_0_ listed in this table are those obtained by fitting the cubes of the lengths of the unit-cell edges; a 2nd-order Birch–Murnaghan EoS was used here as the uncertainties in some of the fitted parameters would otherwise have been very high. The axial EoS for PV-KCaF_3_ allows the macroscopic tilt angle, *Φ*, to be calculated as a function of pressure. When calculated on this basis, *Φ* rises from 10.1° at zero pressure to 16.1° at a pressure of 10 GPa, suggesting either that the relationship proposed by Tateno et al. (2010—see above) does not apply to the ABF_3_ perovskites, or that the minimum value of *Φ*, above which *Φ* will increase with pressure, is less than that for the A^2+^B^4+^O_3_ oxide perovskites. A second feature of the axial EoS shown in Table 2 is the extreme anisotropy of the CaIrO_3_-type phase of KCaF_3_ and the very low incompressibility (8 (2) GPa) of its *b*-axis. As discussed above, the likely mechanism for the formation of the Sb_2_S_3_ and *P*6_3_/*mmc*-NaMgF_3_ phases requires the *b*-axis to be the most compressible so as to reduce the distance between the sheets of BF_6_ octahedra and enable them to cross-link. In KCaF_3_, however, the value of the incompressibility of the *b*-axis is so low as to suggest that the CaIrO_3_ structure may not be stable with respect to formation of the La_2_S_3_, or Sb_2_S_3_ polymorphs, as is indeed found to be the case (see below).

**Table 1:** Equation of state parameters for KCaF_3_, KF and CaF_2_ polymorphs obtained by fitting the internal energy versus volume to integrated 3rd-order Birch–Murnaghan equations of state

	*V*_0_ (Å^3^)	*K*_0_ (GPa)	$K^{\prime}_{0}$	*E*_0_	Volume range fitted (Å^3^)	Transition to next phase (GPa)
KCaF_3_ (PV)	348.62 (6)	44.9167 (5)	4.63 (5)	−104.351 (1)	290–408	5.7 to La_2_S_3_
KCaF_3_ (*P*2_1_/*m*-CaRhO_3_)	336.1 (7)	45.477 (7)	3.36 (7)	−103.68 (1)	320–510	5.4 to Sb_2_S_3_
KCaF_3_ (CaIrO_3_)	347.0 (6)	35.033 (4)	4.03 (5)	−103.72 (2)	185–413	0.23 to La_2_S_3_
KCaF_3_ (La_2_S_3_)	328.7 (8)	48.260 (8)	3.24 (8)	−103.69 (1)	210–340	10.4 to Sb_2_S_3_
KCaF_3_ (Sb_2_S_3_)	316.6 (2)	50.246 (4)	4.47 (6)	−103.115 (2)	230–320	28.3 to *P*6_3_/*mmc*-NaMgF_3_
KCaF_3_ (*P*6_3_/*mmc*-NaMgF_3_)	300.739 (7)	52.1206 (2)	4.709 (4)	−101.0631 (3)	218–336	–
KF (NaCl)	160.24 (9)	29.440 (2)	4.72 (8)	−33.7269 (5)	128–158	7.3 to KF(CsCl)
KF (CsCl)	139.36 (2)	32.9110 (7)	4.83 (2)	−32.9049 (1)	106–140	–
CaF_2_ (fluorite)	168.7 (1)	75.60 (1)	4.8 (3)	−70.307 (1)	148–166	7.9 to CaF_2_ (cotunnite)
CaF_2_ (cotunnite)	155.60 (6)	72.299 (4)	4.85 (8)	−69.6767 (6)	128–148	–

The volume ranges used were chosen so as to eliminate a few higher-pressure data points with larger energy residuals from the fit. The volumes refer to unit cells containing 4 formula units: the *V*_0_ and *E*_0_ parameters for *P*2_1_/*m* have been scaled from 6 to 4 formula units

The dissociation of KCaF_3_ PV to CaF_2_ (fluorite) and KF (NaCl) takes place at 2.62 GPa

The sequences of phase transitions in the simple fluorides were taken from Weir and Piermarini (1964) for KF, and from Wu et al. (2006) for CaF_2_

**Table 2:** Volumetric and axial equation of state (EoS) parameters for KCaF_3_ and CaF_2_ polymorphs obtained by fitting pressure (as output by VASP) versus volume to 2nd-order Birch–Murnaghan EoS (Angel 2001); the volume range used in each case are shown

	Volume		*a*-axis		*b*-axis		*c*-axis	
	*V*_0_ (Å^3^)	*K*_0_ (GPa)	*a*_0_ (Å)	*K*_0_ (GPa)	*b*_0_ (Å)	*K*_0_ (GPa)	*c*_0_ (Å)	*K*_0_ (GPa)
KCaF_3_ (PV) (298–346 Å^3^)	345.36 (7)	48.6 (1)	6.22 (1)	39 (1)	6.301 (4)	62 (2)	8.821 (3)	47.8 (5)
KCaF_3_ (*P*2_1_/*m*-CaRhO_3_) (320–510 Å^3^)	505 (2)	37.2 (6)	14.63 (4)	26 (2)	3.564 (5)	95 (8)	9.81 (1)	38.6 (7)
KCaF_3_ (CaIrO_3_) (276.55–340.45 Å^3^)	341.2 (7)	38.5 (7)	3.47 (1)	73 (6)	12.1 (2)	8 (2)	8.36 (1)	118 (21)
KCaF_3_ (La_2_S_3_) (260–300 Å^3^)	327.3 (2)	43.5 (3)	3.61 (1)	100 (11)	14.42 (5)	21 (1)	6.31 (1)	50 (2)
KCaF_3_ (Sb_2_S_3_) (220–305 Å^3^)	312.4 (3)	56.6 (4)	3.62 (1)	81 (5)	9.46 (4)	34 (3)	9.16 (2)	66 (4)
KCaF_3_ (*P*6_3_/*mmc*-NaMgF_3_) (218.43–298.95 Å^3^)	296.3 (6)	61.6 (8)	4.237 (2)	67.6 (7)	7.337 (5)	68 (1)	9.533 (8)	51.4 (8)
CaF_2_ (cotunnite) (128–152.74 Å^3^)	154.0 (2)	81.3 (9)	6.020 (3)	72 (1)	3.631 (3)	64 (1)	7.06 (2)	117 (4)

To enable ready comparison with the volumetric EoS, the values of *K*_0_ listed in the table are those obtained by fitting the cubes of the lengths of the unit-cell edges. In view of the large uncertainties in some of the fitted parameters the 2nd-order, rather than the 3rd-order Birch–Murnaghan EoS was used. The volumes refer to unit cells containing 4 formula units

So as to enable ready comparison with the CaIrO_3_ and Sb_2_S_3_ structures, the hexagonal *P*6_3_/*mmc*-NaMgF_3_ structure is represented here using a *C*-centred unit cell with an orthorhombic metric in which *b* = √3*a*; the symmetry also requires the value of *K*_*0*_ for these two axes to be the same. In constructing the Table, the *a*- and *b*-axes were fitted independently; the small deviations of the fitted parameters from the relationships expected from the symmetry are not significant

**Fig. 3:**
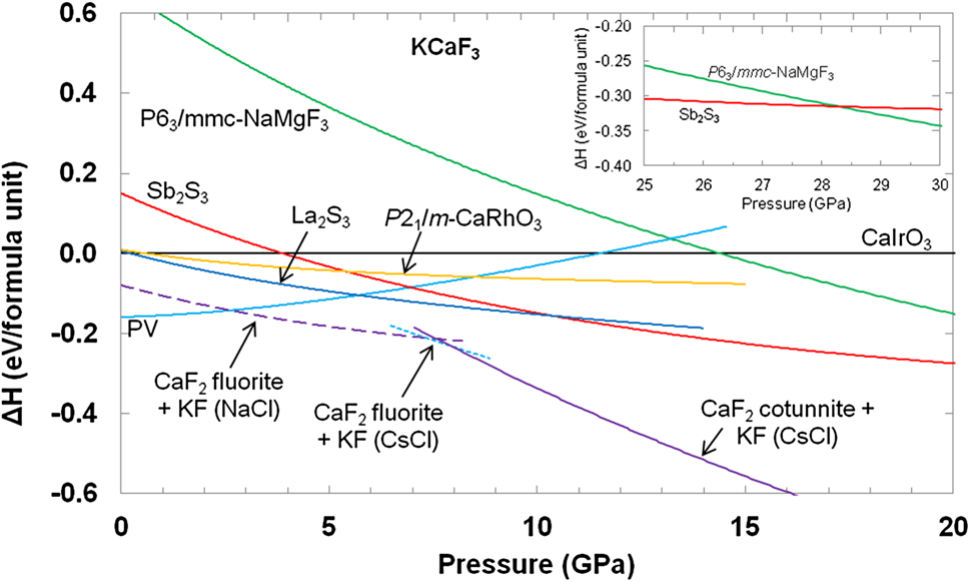
Enthalpies of the PV, *P*2_1_/*m*-CaRhO_3_, La_2_S_3_, Sb_2_S_3_ and *P*6_3_/*mmc*-NaMgF_3_ polymorphs of KCaF_3_ relative to that of KCaF_3_ in the CaIrO_3_ structure. The relative enthalpies of mixtures of the simple fluorides are also shown. The lines for the Sb_2_S_3_ and *P*6_3_/*mmc*-NaMgF_3_ polymorphs cross at 28.3 GPa

**Fig. 4:**
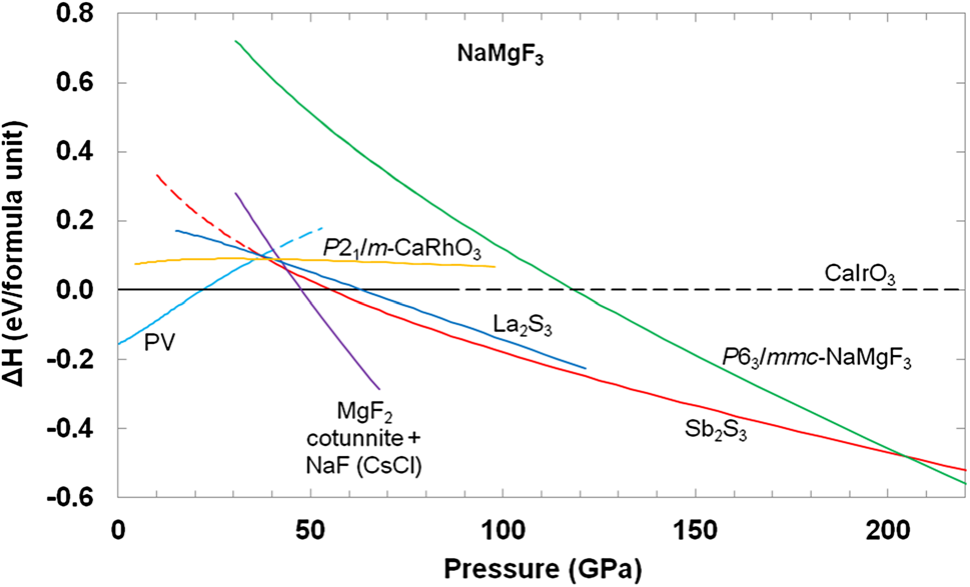
Enthalpies of the PV, *P*2_1_/*m*-CaRhO_3_, La_2_S_3_, Sb_2_S_3_ and *P*6_3_/*mmc*-NaMgF_3_ polymorphs of NaMgF_3_ relative to that of NaMgF_3_ in the CaIrO_3_ structure. The relative enthalpies of mixtures of the simple fluorides are also shown

The enthalpies derived from the EoS parameters listed in Table 1 are shown in Fig. 3, relative to that of CaIrO_3_-structured KCaF_3_. Considering first just the KCaF_3_ polymorphs themselves, it can be seen that a transition from the PV to the La_2_S_3_ structures might be expected at about 5.7 GPa, followed by further transitions to the Sb_2_S_3_ structure at around 10.4 GPa and to the *P*6_3_/*mmc*-NaMgF_3_ structure at 28.3 GPa, confirming our original prejudice that PV-KCaF_3_ might be expected to transform at relatively low pressures. However, Fig. 3 also shows that the CaIrO_3_ structure does not appear to be the relatively most stable polymorph of KCaF_3_ at any pressure. Although we did not expect this result prior to the simulations, it is quite consistent with simple crystal-chemical considerations based on ionic sizes, as the large Ca^2+^ ion is readily able to support coordination numbers higher than six. At atmospheric pressure, the radius ratios of the B cations (in [6]—coordination; Shannon 1976) to the F^−^ ions are 0.541 for Mg/F and 0.752 for Ca/F. The value for NaMgF_3_ thus lies firmly within the range expected for octahedral coordination (0.414–0.732; Pauling 1929). For KCaF_3_, however, the value lies slightly outside this range and just within the range for which a higher coordination number might be expected; on purely geometric grounds, radius ratios in the range 0.732–1.0 are sufficient to support [8]-fold cubic coordination. The CaF_6_ octahedra found in PV-KCaF_3_ must, therefore, be almost at the limit of stability. When pressure is applied to KCaF_3_ and NaMgF_3_ it is to be expected that these radius ratios will increase, as the F^−^ ions will be more compressible than the cations. On compression, therefore, KCaF_3_ may quickly reach the point where octahedral coordination will no longer be stable and thus the La_2_S_3_ and Sb_2_S_3_ structures, in which the coordination will be sevenfold, or almost sevenfold, will form. For NaMgF_3_ (Fig. 4 and see below) this point is not reached until much higher pressure and so there is a stable region of the CaIrO_3_ phase, which has a higher density than the PV phase and in which the structure still contains MgF_6_ octahedra.

However, when the enthalpies of the KCaF_3_ polymorphs are considered relative to that of a mixture of KF and CaF_2_, Fig. 3 also shows that the PV structure is the only polymorph of KCaF_3_ that is ever the thermodynamically most stable phase, and that this is the case only at low pressures. Above about 2.6 GPa, none of the other polymorphs considered are predicted to be stable with respect to dissociation into KF and CaF_2_. The simulations presented here are static and so are effectively athermal, but it seems unlikely that consideration of temperature will significantly affect the thermodynamic stability of the KCaF_3_ phases relative to dissociation into the simple fluorides, since, for example, Fig. 3 shows that by ~7 GPa the enthalpy difference, which increases with increasing pressure, is already about 100 meV per formula unit. It is possible, however, that the high-pressure KCaF_3_ structures could be synthesised by metastable compression in the diamond-anvil cell, since the dissociation reaction requires diffusion of atoms on the multi-unit-cell length scale, which will have a large energy barrier, whereas the structural transitions between the KCaF_3_ phases require no long-range atomic movements and should have much smaller energy barriers. Indeed, the spontaneous transition from the PV to *P*6_3_/*mmc*-NaMgF_3_ structures found in the computer simulations suggests that there is no energy barrier to this transition. Support for this view comes from our recent study of NaCoF_3_ by synchrotron X-ray powder diffraction in a diamond anvil cell, where we found that at 300 K the Sb_2_S_3_ phase of NaCoF_3_ formed at ~23 GPa and persisted to pressures of at least 85 GPa (Wood et al. 2017). In contrast, Yusa et al. (2012) found that, on heating, NaCoF_3_ disproportionated into Na_5_Co_3_F_11_ and NaCo_3_F_7_ at ~20 GPa and 1400 K, with a negative Clapeyron slope, which implies that NaCoF_3_ should not be stable (with respect to other complex stoichiometries) by about 28 GPa at 300 K.

The two unpublished in situ high-pressure experimental studies that have been carried out to date are insufficient to determine whether or not any metastable high-pressure phases of KCaF_3_ exist. In the case of the neutron diffraction study by Knight et al. (2014), it has been confirmed (K S Knight, pers. comm.) that no decomposition products or phase transitions were observed in the range of the experiment, i.e. for pressures ≤6.86 GPa. This pressure is just greater than that at which the transition from the PV to the La_2_S_3_ phase is predicted to occur in our simulations, but when the likely slope of the phase boundary is taken into consideration it may not be sufficient. For NaCoF_3_, the slope of the PV/PPV boundary has been determined to be 15.5 MPa K^−1^ (Dobson et al. 2011); if a similar slope is assumed for the PV/La_2_S_3_ boundary in KCaF_3_, the transition pressure at 300 K rises to ~10.5 GPa, well above that achieved in the neutron diffraction study. Persistence of PV-KCaF_3_ to still higher pressures has been observed ex situ at 300 K in a sample recovered from 15 GPa in our multi-anvil press and also in our in situ diamond-anvil cell study, which showed PV-KCaF_3_ persisting to 20 GPa. However, the fact that no transition to the La_2_S_3_ or Sb_2_S_3_ structures was seen in this experiment does not necessarily mean that the *P*6_3_/*mmc*-NaMgF_3_ structure will not form at higher pressures, as it is the transition from the PV to *P*6_3_/*mmc*-NaMgF_3_ structures that we have observed to occur spontaneously in our computer simulations. Taking account of the slope of the phase boundary, as above, we might expect this transition to occur at ~33 GPa, considerably higher than the maximum pressure of the experiment. We consider, therefore, that a study of KCaF_3_ to ~50 GPa might well be rewarding, since if the *P*6_3_/*mmc*-NaMgF_3_ polymorph of KCaF_3_ does form on further compression we believe that this would be the first occurrence of this structure.

A further possibility would be to determine whether (K, Na)(Ca, Mg)F_3_ solid solutions could provide an alternative route to the various high-pressure structures. Although NaCaF_3_ itself does not appear to exist (at least at atmospheric pressure), doping KCaF_3_ with Na should lead to a substantial reduction in the average size of the A cation, which, in the case of the CaIrO_3_ structure, will produce a substantial reduction in the length of the *b*-axis but only minor changes in the *a*- and *c*-axes. An estimate of the magnitude of these changes can be obtained by considering the CaIrO_3_ structure parameterised in terms of the four shortest cation–anion distances and three tilt angles, as suggested by Lindsay-Scott et al. (2011; Equations 4–6). By putting the interatomic distances equal to the sum of the ionic radii (Shannon 1976), and assuming the same tilt angles as in NaNiF_3_ (Lindsay-Scott et al. 2014), we obtain *a* = 3.48 Å, *b* = 12.12 Å and *c* = 8.67 Å for CaIrO_3_-type KCaF_3_ at *P* = 0, in good agreement with the cell parameters obtained by fitting the axial EoS to the VASP simulations (*a* = 3.47 (1) Å, *b* = 12.1 (2) Å and *c* = 8.36 (1) Å). If the AF distance is now reduced to that appropriate for a composition of K_0.5_Na_0.5_CaF_3_, a value of *b* = 11.00 Å then results (*a* and *c* are unchanged in this approximation), which is equivalent to applying a pressure of ~4.1 GPa. It thus seems quite possible that a combination of applied pressure and chemical substitution might well lead to the formation of the high-pressure KCaF_3_ phases under relatively easily accessible experimental conditions.

### Equations of state for NaMgF_3_ polymorphs and phase diagram of NaMgF_3_

The equation of state parameters for the NaMgF_3_, NaF and MgF_2_ polymorphs are listed in Table 3 and the corresponding enthalpy curves are shown in Fig. 4. Considering first just the NaMgF_3_ phases, it can be seen that the predicted sequence of transitions, with increasing pressure (transition pressures in brackets), is PV to CaIrO_3_ (~22 GPa), CaIrO_3_ to Sb_2_S_3_ (~55 GPa) and Sb_2_S_3_ to *P*6_3_/*mmc*-NaMgF_3_ (~205 GPa). Our results are, therefore, in good accord with those of Umemoto and Wentzcovitch (2006) who found the same sequence of transitions, occurring at about 18, 44 and 223 GPa. Conversely, Xu et al. (2015) found the sequence PV to CaIrO_3_ (~19 GPa), followed by CaIrO_3_ to La_2_S_3_ (~51 GPa). All three studies agree in that they indicate a fairly wide phase field (~30 GPa) for the CaIrO_3_ structure, reflecting the ability of the Mg ions to remain in octahedral coordination to higher pressures (see above). Although the stable phase at pressures above ~55 GPa predicted by Xu et al. (2015) differs from the Sb_2_S_3_ structures found by Umemoto and Wentzcovitch (2006) and in the present study, it should be pointed out that the difference in enthalpy is small, being less than 10 meV/atom, and so the sequence of phases might differ at finite temperatures. There is, however, now strong experimental evidence, including crystal structure refinements, for the formation of Sb_2_S_3_ structures in some ABF_3_ compounds, as essentially phase-pure samples have been reported for NaFeF_3_ by Crichton et al. (2016) and for NaCoF_3_ by Wood et al. (2017). The experimental evidence for the formation of the La_2_S_3_ structure is, at present, much weaker. Xu et al. (2015) considered that their simulated powder diffraction pattern of La_2_S_3_-structured NaMgF_3_ resembled that of the “N phase” reported by Martin et al. (2006a), but the space group and unit-cell parameters suggested by Martin et al. are different. Martin et al. (2006a) have space group *Pnnm* and experimental unit-cell parameters of *a* = 8.353 (3) Å, *b* = 5.265 (2) Å, *c* = 5.857 (3) Å, *V* = 257.58 (3) Å^3^ at 37 (1) GPa, whereas Xu et al. (2015) have space group *Pnma*, with *a* = 5.082 Å, *b* = 2.829 Å, *c* = 10.215 Å, *V* = 146.9 Å^3^ for their simulation of the La_2_S_3_ structure at 60 GPa. Correspondence of the “N phase” of Martin et al. (2006a) with the La_2_S_3_ structure would, therefore, seem to imply that the experimental powder pattern has been mis-indexed.

**Table 3:** Equation of state parameters for NaMgF_3_, NaF and MgF_2_ polymorphs obtained by fitting the internal energy versus volume to integrated 3rd-order Birch–Murnaghan equations of state

	*V*_0_ (Å^3^)	*K*_0_ (GPa)	$K^{\prime}_{0}$	*E*_0_	Volume range fitted (Å^3^)	Transition to next phase (GPa)
NaMgF_3_ (PV)	235.73 (6)	69.801 (2)	3.94 (2)	−99.1497(8)	175–235	21.9 to CaIrO_3_
NaMgF_3_ (*P*2_1_/*m*-CaRhO_3_)	234.6 (2)	57.557 (2)	4.55 (1)	−98.281(5)	140–220	38.7 to Sb_2_S_3_
NaMgF_3_ (CaIrO_3_)	232.38 (7)	61.504 (1)	4.49 (1)	−98.527(1)	145–234	55.1 to Sb_2_S_3_
NaMgF_3_ (La_2_S_3_)	233.2 (6)	55.635 (6)	4.52 (3)	−97.77(3)	130–195	36.7 to Sb_2_S_3_
NaMgF_3_ (Sb_2_S_3_)	218.7 (3)	78.153 (4)	4.31 (1)	−96.55(3)	105–170	204.7 to *P*6_3_/*mmc*-NaMgF_3_
*P*6_3_/*mmc*-NaMgF_3_	211.9 (3)	84.751 (4)	4.270 (8)	−93.96(2)	100–170	–
NaF (CsCl)	93.96 (4)	49.7683 (8)	4.524 (3)	−33.336(2)	49–71	–
MgF_2_ (cotunnite)	119.6 (1)	86.542 (7)	4.83 (5)	−61.350(6)	85–105	–

The volumes refer to unit cells containing 4 formula units: the *V*_0_ and *E*_0_ parameters for *P*2_1_/*m* have been scaled from 6 to 4 formula units

The dissociation of CaIrO_3_-type NaMgF_3_ to MgF_2_ (cotunnite) and NaF (CsCl) takes place at 47.6 GPa

When the enthalpies of the NaMgF_3_ polymorphs are considered relative to that of a mixture of NaF and MgF_2_, however, Fig. 4 shows that only the PV and CaIrO_3_ structures will be the thermodynamically most stable phases. For pressures above ~48 GPa we find that all NaMgF_3_ polymorphs are unstable with respect to dissociation into NaF and MgF_2_. Once again our results are in accord with the original findings for NaMgF_3_ of Umemoto and Wentzcovitch (2006) who reported a dissociation pressure of ~40 GPa.

Our findings for both NaMgF_3_ and KCaF_3_, therefore, support the view expressed previously by Umemoto et al. (2006a), Grocholski et al. (2010) and Tsuchiya and Tsuchiya (2011) that in the natural silicate system as found in the deep interiors of the mantles of large terrestrial exoplanets or in the cores of gas giants, dissociation into high-pressure polymorphs of the simple oxides, MgO and SiO_2_, will occur before the formation of any of the high-pressure structures considered here.

## Footnotes

1 In this context, the term “perovskite (PV)” refers to the GdFeO_3_-type structure (as found in the mineral bridgmanite; Tschauner et al. 2014) and the term “post-perovskite (PPV)” refers to the CaIrO_3_ structure (Rodi and Babel 1965).

2 Crichton et al. (2016) refer to this structure as being identical to the α-Gd_2_S_3_ type, but in the Inorganic Crystal Structure Database (ICSD; Fletcher et al. 1996) this type structure is currently given as La_2_S_3_.

3 Following the “perovskite > post-perovskite” terminology it is tempting to refer to any higher-pressure phases as “post-post-perovskite” or even “post-post-post-perovskite”, as has been done in the past by others. However, acting on admirable advice from Wilson Crichton (pers. comm.) and an anonymous reviewer, we now believe that it is far better to use structure-type labels whenever possible. The succession of phases following the perovskite structure at high pressures has now been found to differ for different ABX_3_ compounds (e.g. Shirako et al. 2012a; Xu et al. 2015) and so there can now be confusion as to what is implied by, e.g. a “post-post-perovskite structure”. We have, though, retained the term “perovskite” instead of “GdFeO_3_-type” as this term is in such common usage. Similarly, in the case of MgSiO_3_, the term “post-perovskite” has been retained as indicating the CaIrO_3_-type structure. One of the structures that we consider here, that with space group *P*6_3_/*mmc* (Umemoto and Wentzcovitch 2006) does not seem to have been observed experimentally and so we refer to this as the “*P*6_3_/*mmc*-NaMgF_3_ structure”. Similarly, we refer to the monoclinic (*P*2_1_/*m*) “intermediate phase” of Shirako et al. (2012a) as the “*P*2_1_/*m*-CaRhO_3_ structure”, as this has no structure type in the ICSD (Fletcher et al. 1996).
